# Artificial Intelligence in Spinal Imaging: Current Status and Future Directions

**DOI:** 10.3390/ijerph191811708

**Published:** 2022-09-16

**Authors:** Yangyang Cui, Jia Zhu, Zhili Duan, Zhenhua Liao, Song Wang, Weiqiang Liu

**Affiliations:** 1 Tsinghua Shenzhen International Graduate School, Tsinghua University, Shenzhen 518055, China; 2 Department of Mechanical Engineering, Tsinghua University, Beijing 100084, China; 3 Biomechanics and Biotechnology Lab, Research Institute of Tsinghua University in Shenzhen, Shenzhen 518057, China

**Keywords:** artificial intelligence, image presentation, deep learning, image interpretation, machine learning, spinal imaging

## Abstract

Spinal maladies are among the most common causes of pain and disability worldwide. Imaging represents an important diagnostic procedure in spinal care. Imaging investigations can provide information and insights that are not visible through ordinary visual inspection. Multiscale in vivo interrogation has the potential to improve the assessment and monitoring of pathologies thanks to the convergence of imaging, artificial intelligence (AI), and radiomic techniques. AI is revolutionizing computer vision, autonomous driving, natural language processing, and speech recognition. These revolutionary technologies are already impacting radiology, diagnostics, and other fields, where automated solutions can increase precision and reproducibility. In the first section of this narrative review, we provide a brief explanation of the many approaches currently being developed, with a particular emphasis on those employed in spinal imaging studies. The previously documented uses of AI for challenges involving spinal imaging, including imaging appropriateness and protocoling, image acquisition and reconstruction, image presentation, image interpretation, and quantitative image analysis, are then detailed. Finally, the future applications of AI to imaging of the spine are discussed. AI has the potential to significantly affect every step in spinal imaging. AI can make images of the spine more useful to patients and doctors by improving image quality, imaging efficiency, and diagnostic accuracy.

## 1. Introduction

Imaging is still used to evaluate patients with spinal disorders, and its utility has contributed to a rise in the use of popular spinal imaging modalities [1]. Increased utilization has created multiple challenges for a radiology department or private practice, including a greater demand for operational efficiency while maintaining good accuracy and imaging report quality [2]. As seen by the dramatic rise in the number of published articles over the past few years, AI is increasingly being utilized to explore spine-related issues [3,4,5], particularly in radiological imaging but also in other disciplines such as treatment outcome prediction. In a number of applications, the reported findings are either promising or have already surpassed the prior state of the art; for instance, AI approaches now enable an exact and fully reproducible grading of intervertebral disc degeneration on magnetic resonance imaging (MRI) scans [6]. Indeed, the current rate of technological advancement is anticipated to yield additional benefits in the near future. Radiologists can use AI as an innovative tool to meet these demands. AI has the potential to have a substantial impact on each step of the imaging value chain. At this early stage of the integration of AI into radiology, some studies using spinal imaging have previously investigated and shown the potential utility of AI [7,8]. The purpose of this article is to introduce artificial intelligence (AI) to spinal radiologists through a review of recent research that emphasizes AI’s use at various phases of spinal image production and utilization. We anticipate that, in the future, spinal imaging will be performed using AI.

With this narrative literature review, we seek to elucidate AI’s existing successes and potential spine-related applications for scientists in the field as well as readers from other domains who are unfamiliar with the technical elements of such technologies. To achieve this objective, the paper begins with a brief summary of AI’s practical or potential impact on spinal research. Image capture and reconstruction, image presentation, image interpretation, and quantitative image analysis, as well as determining the appropriateness of imaging orders and predicting patients at risk for fracture, are described in the following sections.

## 2. Technical Aspects

Despite the fact that the phrases “AI”, “machine learning” (ML), and “deep learning” (DL) are sometimes used interchangeably, there are major distinctions in what each of these related terms means. The term “AI” refers to any method that can teach computers to behave like intelligent humans [9]. ML is a highly specialized branch of AI that uses a variety of tools derived from statistics, mathematics, and computer science to enable machines to improve their performance in jobs as they gain more experience. DL is a subset of ML that explores the application of a specific category of computer models known as deep neural networks to big datasets. This subfield is even more specialized than traditional ML. DL [10] has resulted in a number of ground-breaking advancements in a variety of fields, including image classification [11] and semantic labeling [12]. This is primarily attributable to the rapid development of neural networks, which are mathematical or computer models that mimic the structure and function of biological neural networks (the central nervous system of animals, especially the brain) [13]. Neural networks consist of a large number of artificial neurons made using a variety of connection techniques. The Convolutional Neural Network (CNN) is one of them, along with Generative Adversarial Networks (GANs), Recurrent Neural Networks (RNNs), etc. Neural networks can have the same simple decision-making ability and simple judgmental ability, and can produce superior results in image and speech recognition [14,15].

With improved research and application potential, AI technology has gradually become more standardized and disciplined. As a multidisciplinary science, artificial intelligence technology focuses on machine anthropomorphic recognition, learning, and thinking, so its technical content is expanding in the development process, including knowledge graphs, intelligent optimization algorithms, expert systems, machine logic, and other research content [16,17]. A knowledge graph is a collection of graphs that depict the evolution of knowledge and its structural relationships. It describes knowledge resources and their carriers using visualization technology, and mines, analyzes, constructs, draws, and displays knowledge and its relationships [18]. Intelligent optimization algorithms include genetic algorithms, ant colony algorithms, tabu search algorithms, simulated annealing algorithms, particle swarm algorithms, and others. Intelligent optimization algorithms are typically built for specific situations with few theoretical prerequisites and a high level of sophistication. Usually, intelligent algorithms are compared with optimization algorithms. Intelligent algorithms, on the other hand, are rapid and have a wide range of applications [19]. An expert system is a computer program system that has a substantial quantity of knowledge and expertise at the expert level on a specific topic. It can use artificial intelligence and computer technology to reason and judge based on the system’s knowledge and experience, emulating human experts. An expert system is a computer program system that simulates human experts to solve domain problems in order to address complicated problems that human experts need to manage [20]. Currently, it is also utilized extensively in big data and IoT systems [21,22].

At present, the data-driven ML process has been derived into a fully automated program that can process large amounts of data without manual intervention. DL is a new method of ML that breaks through the bottleneck of traditional ML methods. Compared to conventional ML, DL deep networks scale better with more data, require no feature engineering, and are easily transformable [23]. In many fields, including voice, natural language, vision, and game playing, deep neural networks have surpassed classic ML techniques in terms of accuracy. In many situations, classical ML cannot even compete with DL [24]. Through multi-layer processing, the initial low-level feature representation is gradually transformed into a high-level feature representation, and features can be automatically identified to complete complex classification and other learning tasks. Fully automatic data analysis has become a reality, which has accelerated the growth of ML. Research shows that DL can provide more personalized and precise treatment plans [25,26]. The most basic method of ML is to use algorithms to parse data, learn from that data, and then make decisions and predictions about events in the real world. Compared with traditional ML methods that are manually designed with features, the DL method based on big data drives the learning of image features from the image itself, rather than from experience, so one can learn the features of the image more comprehensively. In the face of high-dimensional nonlinear data, DL methods can adaptively learn appropriate features and finally classify images with high accuracy. Therefore, in recent years, they have received extensive attention from academia and industry.

The CNN is one of the most essential architectural types when processing image data [27]. Each node in a CNN is only connected to a small number of nearby nodes. This structure is highly effective in extracting local image features (each node is only connected to a small number of nearby nodes). This structure is particularly effective in extracting local image features (each node is only connected to a small number of nearby nodes). A CNN is a typical model used in DL applications to extract image features. It is an end-to-end network model type. It only takes the input of the image with category labels, and the network can automatically execute hierarchical learning of image characteristics and learn deeper image features by appropriately increasing the number of network layers. This structure is particularly effective in extracting local image characteristics Inception-v4 was introduced by Szegedy et al. [28]. The residual structure is integrated into the Inception network to improve training efficiency, and two network structures, Inception-ResNet-v1 and Inception-ResNet-v2, are proposed to further promote the application of a CNN. Based on the existing CNN structure, Ronnenberge et al. [29] constructed a more elegant architecture, the so-called “fully convolutional network.” Modifying and extending this architecture so that it can work on few training images and produce more accurate segmentations has also advanced the use of CNNs on medical images.

## 3. Imaging Appropriateness and Protocoling

Making the best imaging examination choice for a patient can be challenging for a doctor. Although there are resources that can help with this issue, such as decision support software, virtual consult platforms, and imaging ordering recommendations, ML can offer a more thorough, evidence-based resource [30,31]. In addition, a significant corpus of research is also starting to exploit multimodal, multiscale data fusion for biomedical imaging and ML applications [32]. In order to recommend an appropriate patient-specific imaging examination tailored to the clinical question that must be answered, ML algorithms can incorporate multiple sources of information from a patient’s medical records, such as symptoms, laboratory values, physical examination findings, and previous imaging results [33]. After the requisite examination has been ordered, it is the radiologist’s obligation to guarantee that it is properly protocoled and executed. Inadequately protocoled investigations can result in inferior patient treatment and outcomes, repeat examinations that may require more radiation exposure, significant aggravation and difficulty for both patients and referring physicians, and an increase in the radiology practice’s expenses.

Recently, there have been two studies that have investigated the application of DL for natural language classification as well as its possible utility in automatically establishing spine-related protocols and the necessity of IV contrast media [34,35]. The research conducted by Lee [34] demonstrated that it is possible to use deep CNNs to differentiate between spinal MRI examinations that follow routine protocols and those that follow tumor protocols. The researchers used word combinations such as “referring department”, “region”, “contrast media”, “gender”, and “age” in their investigation [33]. Based on the free-text clinical indication, Trivedi et al. [35] employed a DL-based natural language classification system (Watson, IBM) to assess whether or not an IV contrast medium was required for spinal MRI tests. Even though both studies found promising results for potential applications in clinical decision making and protocoling support, there is no doubt that future research will investigate increasingly complex classifiers in order to more accurately reflect the variety of spinal imaging protocols that are currently available. For instance, ML could use the information on the examination order as a starting point, but it could also potentially mine electronic medical records, prior examination protocols and examination reports, Computed Tomography (CT) or Magnetic Resonance Imaging (MRI) scanner data, contrast injection system and contrast agent data, cumulative or annual radiation dose information, and other quantitative data [36]. This would allow ML to determine which protocol would be the most appropriate.

## 4. Image Acquisition and Reconstruction

### 4.1. Increase the Speed of Medical Imaging

Imaging speed has always been one of the important factors that have attracted much attention in the process of clinical medical imaging scanning. Excessive scanning time will reduce the daily average circulation of imaging departments and cause discomfort to patients. In terms of rapid imaging, relevant international research mainly focuses on the acceleration of magnetic resonance imaging. Mardani et al. [37] proposed a magnetic resonance (MR) compression based on a GAN compressed sensing (CS) rapid imaging method, which uses a GAN to model the low-dimensional manifold of high-quality MR images. The GAN was introduced to the research community by Dr. Goodfellow [38]. A GAN is composed of a generator and a discriminator. The function of the generator is to map low-quality MR images onto a manifold of high-quality images. In order to ensure that the generated images are true and reliable, the author introduces the k-space data fidelity item into the network. Experimental results show that this method can achieve at least 5 times the scanning acceleration, and the imaging results are significantly superior to those of traditional compressed sensing algorithms.

Schlemper et al. [39] proposed a fast MR imaging method based on a cascaded DNN. The cascaded deep neural network is formed by the cascade of several network units. Each network unit contains two parts: a CNN and a data fidelity item. A CNN is a residual network (residual network, Res-Net) from construction. The experimental results show that the reconstructed image quality of the cascaded deep neural network is significantly improved compared to the traditional compressed sensing method and the image reconstruction method based on dictionary learning. At the same time, it takes only 23 ms to reconstruct a two-dimensional image of the spine, which basically achieves a quasi-real-time effect. The quasi-real-time effect means that the processing can be completed in a very short time. This time can be ignored, so it can be understood as a real-time effect. In order to further improve the quality of the reconstructed image, Chen et al. [40] proposed a multiecho image joint reconstruction method, which uses U-Net [41] to achieve image reconstruction. Taking the 6-echo image as different input channels makes it possible to make full use of the structural similarity between different echo images in the convolution process, thereby adding more constraints to the training of the network and making the training process more stable. Experimental results show that this method can achieve 4.2 times faster MR imaging, and the reconstructed image is better than the single contrast reconstruction method in terms of the root mean square error (RMSE) and the structural similarity index (SSIM). Due to the spatial local characteristics of CNN convolution operations, most of the fast-imaging methods based on DL currently process in the image domain. However, some image artifacts caused by the incompleteness of k-space data are difficult to solve perfectly in the image domain. In order to solve this problem, Taejoon et al. [42] proposed a fast MR imaging method based on dual-domain DL. Corresponding deep CNNs were designed in both the image domain and the frequency domain, and they attempted to perform the imaging in two different spaces. The uncollected data is restored, and the image domain and the frequency domain are correlated through data fidelity items, thereby ensuring the reliability of the reconstructed image. The experimental results show that the roles of the image domain CNN and frequency domain CNN in the image reconstruction process are different. Compared with the imaging method that only uses the image domain CNN, the combination of the two can obtain higher quality image reconstruction results.

### 4.2. Decreasing CT Radiation Doses

Diagnostic imaging patients are exposed to a significant amount of radiation. In this study, ionizing radiation, not heat or electromagnetic radiation, is the focus. However, there have been ongoing efforts [43,44] to reduce this dose. The use of AI offers a promising new method for lowering the radiation dose required for CT scans. The current AI-based techniques for radiation reduction function in a manner that is comparable to that of techniques used to increase the speed of MRI acquisition. More specifically, the goal of these techniques is to reconstruct high-quality images using fewer raw data points or raw data points of lower quality. Wolsterink et al. [45] published a study in 2017 in which they used a GAN to predict conventional-dose CT images from low-dose CT images. As a result, the noise in the low-dose CT images was reduced. More than 90% of readers in a recent study [46] by Cross et al. found that the quality of low-radiation-dose CT images, which were produced in part with the use of an artificial neural network (ANN), was equal to or greater than that of CT images obtained using standard radiation doses. This conclusion was reached based on the findings of the study that was conducted by Cross et al.

## 5. Image Presentation

### 5.1. The Intelligent Workflow of Spinal Imaging

Radiologists are under ever-increasing pressure to boost productivity, as they are asked to interpret greater daily quantities of more difficult cases than in the past [47]. If the Picture Archiving & Communication System (PACS) automatically shows each series in the correct chosen position, orientation, and magnification, as well as the correct preferred window and level, syncing, and cross-referencing settings, radiologists can work more efficiently. Such hanging protocols should be uniform and based precisely on modality, body part, laterality, and time (in the case of prior available imaging). By employing smarter technologies that process a range of data, AI has the potential to transform the way a PACS displays information to a radiologist. One PACS provider employs ML algorithms to discover how radiologists prefer to watch examinations, collect contextual data, present layouts for future similar studies, and modify the following corrections [48]. These intelligent solutions can assist radiologists in achieving improved productivity by resolving challenges associated with fluctuating or missing data that might cause traditional hanging methods to fail.

In recent years, the rapid development of AI technology has gradually enabled an intelligent spinal imaging scanning workflow. This intelligent scanning workflow covers functions such as patient identity intelligent authentication, intelligent voice interaction, intelligent patient positioning, and intelligent scanning parameter setting throughout the entire image scanning process, and its purpose is to significantly reduce the repetitive work of scanning physicians and improve the circulation of patients in the hospital, which improves the medical experience of patients and, at the same time, increases the consistency of the image data collected by different physicians.

At present, there is little research work in the field of intelligent workflow in academia. Existing work mainly focuses on intelligent scanning and positioning. Among them, the fast and accurate automatic positioning of human anatomy is the core of its function. Kelm et al. [49] proposed an automatic positioning method for human anatomical structures called marginal space learning (MSL), which modeled the anatomical structure positioning as a search process for specific anatomical structures in medical images. The search space (including dimensions such as position, size, and angle) is considerable, making the time consumption of exhaustive search methods unacceptable. The principle of MSL is to prune the impossible situations in advance during the search process, thereby avoiding a large number of useless searches. The effective search space is only a small part of the complete search space, so it is called edge space learning. MSL has a wide range of applications and can realize the rapid positioning of different human anatomical structures. This reference introduces the experiment of using MSL to automatically locate the spine in MR images. The results show that the CPU version of the MSL algorithm can detect all lumbar intervertebral discs within an average of 11.5 s, with a sensitivity of 98.64% and a false positive rate of only 0.0731, which has high clinical application value.

In addition to the automatic positioning of tissues and organs, the automatic positioning of key points (landmarks) is also important in the intelligent scanning workflow. Most of the existing methods first learn a feature model of structure and texture and then search for the key points of interest in the image based on this model. Usually, these feature models are calculated based on the local information of the image, which makes it easy to fall into the local extremum. In order to solve the above-mentioned problems, Ghesu et al. [50] proposed a novel key point location method, which treats the key point feature modeling process and search process as a unified process. Specifically, this method uses DL methods to achieve multi-level image feature extraction and uses reinforcement learning (RL) methods to achieve efficient spatial search, and the deep neural network is used to combine the two together to perform the end-to-end learning process and effectively improve the overall detection effect of the algorithm. This reference has conducted algorithm tests on two-dimensional MR images, two-dimensional ultrasound images, and three-dimensional CT images. The experimental results show that the algorithm is far superior to the existing key point detection algorithms in accuracy and speed, with an average error of 1~2 pixels. When the key point does not exist, the algorithm can automatically give corresponding prompts, which has a wide range of applications and good practical value. Aiming at 3D CT and MR images, Zhang et al. [51] proposed a fine-grained automatic recognition method of human body regions. Compared with the computer vision field, the labeled data in the medical imaging field is relatively small. Transfer learning can usually be used in order to solve the problem of network training over-fitting. However, there is a substantial difference between natural images and medical images, so transfer learning based on natural images cannot achieve optimal results in many cases. The innovation of the method proposed in this reference is that a self-supervised network transfer learning method is designed, such that CT or MR images themselves can be used for self-learning, thereby avoiding images in different fields of problems caused by large differences. The experimental results in their study show that, compared to the cross-domain transfer learning from natural images to medical images, the label-free self-supervised transfer learning in the domain proposed in this reference can obtain significantly better recognition results [51].

In the industry, work related to an intelligent scanning workflow has been reported. Germany’s Siemens AG has developed a fully assisting scanner technology (FAST) system, which uses high-precision 3D cameras to achieve accurate patient positioning. In particular, the 3D camera can obtain a three-dimensional contour of the patient’s body using infrared light technology, calculating the patient’s body shape and other useful information based on this information, and performing other functions such as automatic isocenter positioning and automatic scanning range as a result. The consistency of picture scanning is improve, and unwanted radiation exposure is lowered. Generally speaking, the research and development in the field of medical imaging intelligent scanning workflow is still in its infancy, and breakthroughs and innovations have been sporadic. The entire imaging scanning chain has not yet been fully opened, and many innovative researches with clinical value need to be continued in the future, so as to improve the patient’s diagnosis and treatment effect and medical experience and reduce the heavy and repetitive workload of the scanning physician.

### 5.2. The Quality Enhancement of Medical Images

#### 5.2.1. CT Image Quality Enhancement

Based on Section 4.2, the enhancement research on CT image quality mainly focuses on how to use AI technology to manage the noise caused by the reduction of radiation doses and the streak artifact caused by the reduction of the number of projections. In terms of low-dose image denoising, Chen et al. [52] proposed a CT image denoising method based on a residual autoencoder, which uses a deep neural network to construct an autoencoder. Compared with traditional image denoising methods, Li et al. [53] designed a GAN-based CT image denoising method. The GAN is used to learn the mapping from low-dose images to normal-dose images, and the discriminating network is used to determine whether the generated denoised images are in the manifold where the normal dose image is located, i.e., whether it is visually similar to the real normal-dose image. The experimental results show that the GAN-based image denoising method proposed by Wolterink et al. can effectively remove the noise in low-dose CT images and, at the same time, well preserve the details of the image, increasing the visual credibility of the denoised image. In addition, there are related studies on the suppression of streak artifacts in CT images by deep neural networks. The noise in low-dose images is usually local, but the streak artifacts caused by sparse projection sampling are global, so a larger receptive field is needed when constructing the network. Han et al. [54] proposed an algorithm for removing streak artifacts based on U-Net, which is different from other algorithms used for removing artifacts. The Net method is inadequate for handling streak artifacts; specific improvement strategies were presented, and the dual-frame U-Net and tight-frame U-Net were proposed by the authors. The experimental results show that the artifact suppression effect of the two improved networks is significantly stronger than the classic U-Net network, and the details of the anatomical structure are more complete. In some cases, due to physical and mechanical constraints, only CT projection data within a certain angle range can be obtained. When traditional analytical reconstruction methods and iterative reconstruction methods are used for this type of data incompleteness problem, the reconstructed image usually contains serious artifacts and blur. To solve this problem, Anirudh et al. [55] proposed a limited-angle CT image de-artifacting algorithm (CT-Net) based on DL. The basic idea is to directly learn the mapping from an incomplete sinogram to a CT image in the training process of CT-Net. In order to ensure that the enhanced image has a greater signal-to-noise ratio and rich detailed information, the loss function integrates the image domain L2 norm and the GAN. In the application process, CT-Net is first used to obtain the enhanced CT image, the image is then used to fill the missing chord diagram, and the analytical or iterative reconstruction method is finally used to reconstruct the final image using the completed chord diagram. In the experiment, the author obtained only 90° chord diagram data; nonetheless, utilizing CT-Net, high-quality images could be reconstructed, and traditional analytical or iterative reconstruction methods cannot produce such unambiguous findings.

#### 5.2.2. PET Image Quality Enhancement

Due to the fact that PET imaging involves the injection of radioactive tracers (such as 18F-FDG) into the patient’s body in advance to minimize the radiation dose received by the patient, there is a significant clinical demand for low-dose PET imaging; yet, the reduction in doses will generate image noise. Increasing and decreasing, in contrast, have an impact on the clinical diagnosis of illnesses. In response to this issue, Xu et al. [56] suggested a residual encoder–decoder-based PET image enhancing approach. Compared to classic non-local means (NLM) and block-matching and 3D filtering (BM3D) methods, the method described by the authors can achieve high quality at a normal dose of 0.5%. At the same time, the processing time for a 2D PET image is only 19 ms, which is far less than the processing time required by traditional methods.

#### 5.2.3. MR Image Quality Enhancement

In order to achieve imaging acceleration, data truncation and zero-filling are usually performed in k-space, which will cause Gibbs artifacts in the reconstructed image. Traditional MR image artifact removal methods are usually based on k-space filtering, but k-space filtering cannot distinguish between artifact signals and useful signals, so the enhanced image often has problems such as excessive smoothness and a loss of details. In order to solve this problem, Neusoft Medical proposed an MR image enhancement method based on multi-task learning (MTL) [57], which is based on U-Net and ResNet network structures, which can realize Gibbs artifact suppression. Experiments show that the MTL-based MR image enhancement method can effectively suppress Gibbs artifacts while protecting the image resolution.

## 6. Image Interpretation

Detecting patterns and interpreting images is now the most prominent field of research in ML. ML algorithms have been applied to a variety of disorders, including lumbar degenerative disease, scoliosis, spinal malignancies, spinal cord compression, cervical spondylosis, and osteoporosis.

### 6.1. Lumbar Degenerative Disease

In 2017, Azimi et al. [58] reported the use of neural networks for decision-making assessment of lumbar spinal stenosis, using an ANN model and an LR model to predict and analyze 346 patients. Compared with the LR model, the ANN model shows a higher accuracy (97.8%), improved Hosmer and Lemeshow statistics (41.1%), and has a higher area under the curve (AUC) of 89%. In 2018, Han et al. [59] used a multi-modal and multi-task DL model to simultaneously locate and grade multiple spinal structures (intervertebral foramina, nerve root canal, and intervertebral disc), as well as diagnose lumbar spinal stenosis based on automatic pathogenesis, and found the causative factors; they proposed a multi-modal multi-task learning network, by merging semantics, expanding the multi-modal convolutional layer to expand multiple output layers, and adding multiple task regression loss, and finally achieved an average accuracy of 84.5% in the MRI, T1, and T2 weighted images of 200 subjects. In 2017, Kim et al. [60] proposed an ANN model that can accurately predict incision complications, venous thromboembolism, and mortality after lumbar posterior fusion. The study included 22,629 cases; 70% were used in the training set, and 30% were used in the test set. The predictors included information such as gender, age, race, diabetes, smoking, hormones, and coagulation dysfunction. The result was an ML model in the form of an ANN, which suggests that the risk factors for lumbar fusion surgery have higher sensitivity and accuracy than other AI models. In 2018, Staartjes et al. [61] proposed that, based on the prognosis of patients after lumbar discectomy, a prediction model based on morbidity and logistic regression was established for preoperative prediction. A total of 422 patients were included, and an 85% prediction accuracy of the recurrence rate after lumbar discectomy was obtained, which leads to the possibility of informing patients of symptom improvement before surgery.

### 6.2. Scoliosis

In addition to the degenerative spine, AI approaches have also been applied to the investigation of spinal abnormalities. Evaluation of the severity of adolescent idiopathic scoliosis using noninvasive techniques, such as surface topography, is the research field that has been most influenced by AI. In fact, these techniques do not provide a direct image of the spine; consequently, the extraction of clinically relevant conclusions can benefit greatly from inference methods that can exploit subtle patterns in the data that may not be obvious to human observers.

Ramirez et al. [62] categorized the surface topographies of scoliotic patients into three groups, namely, mild, moderate, and severe curves, using a support vector machine, a decision tree, and linear discriminant analysis. The authors attained an 85% accuracy with the support vector machine (SVM), which outperformed other classifiers. Bergeron et al. [63] utilized an SVR to extract the spinal centerline from surface topography, employing biplanar radiographs of 149 scoliotic individuals as ground truth data. The first attempt to predict the curve type, a simplified version of the Lenke [64] classification system distinguishing three types of scoliotic curves, was performed by Seoud et al. [65], who used an SVM trained on radiographs of 97 adolescents suffering from idiopathic scoliosis and achieved an overall accuracy of 72.2% with respect to diagnoses based on measurements performed on planar radiographs. Komeili et al. [66] trained a decision tree to classify surface tomography data into mild, moderate, and severe curves, and to identify the curve location in order to assess the risk of curve progression. The model was capable of detecting 85.7% of the progression curves and 71.6% of the non-progression curves.

Using AI approaches, the analysis of radiographic data from patients with spinal abnormalities has also been attempted. The difficult automated analysis of the Cobb angle representing the severity of a scoliotic curve has been tackled in a variety of ways, ranging from non-ML techniques such as the fuzzy Hough transform to DL techniques [67]. By extracting 78 landmark points, Galbusera et al. [68] suggested a new DL method for X-ray analysis of scoliosis (such as the center of the upper and lower endplates, the center of the hip joint, and the edge of the S1 endplate). A patient’s spine underwent three-dimensional reconstruction, and a new convolutional neural network capable of simulating various spine configurations with an error range of 2.7–11.5° was developed. It was shown that this approach can automatically identify the spine in X-rays. The scoliosis’s shape is calculated. Zhang et al. [69] trained a deep ANN to predict the vertebral slopes on coronal radiography images and used the slope data to estimate the Cobb angle, with absolute errors below 3°. Wu et al. [70] utilized the three-dimensional information available in biplanar radiographs to conduct a more exhaustive evaluation of the abnormal curvature. Thong et al. [71] aimed to create a novel classification scheme for teenage idiopathic scoliosis that effectively describes the variety of the curves among the participants by employing an unsupervised clustering technique.

### 6.3. Spinal Tumors

At the beginning of 2019, Bi et al. discussed the current status, prospects, and challenges of the application of AI in the field of cancer. Most of the AI used in oncology has not been vigorously verified for repeatability and universality, but these studies still promote the clinical use of AI and affect the future direction of cancer treatment [72]. In 2017, Wang et al. [73] applied a conjoined deep neural network to automatically identify spinal metastases in MR images and developed a conjoined deep neural network method that includes three identical sub-networks. It is used to analyze spinal metastases under multi-modality; an evaluation of 26 cases shows that the accuracy of the proposed method to correctly detect spinal metastases is 90%, which reaches clinical requirements. In 2019, the team further reported the image identification of lung cancer spinal metastases based on dynamic contrast enhanced magnetic resonance imaging (DCE-MRI), extracting histograms and texture feature parameter maps from three DCEs and using them as input to train CNNs and convolutional long short-term memory (CLSTM) networks. The final accuracy of the CNN is 71 ± 4.3%, while the accuracy of the CLSTM network is 81 ± 3.4% [74]. In 2018, Karhade et al. [75] reported that the American College of Surgeons developed an ML algorithm to predict the 30-day mortality rate after spinal metastasis, and selected the algorithm with the best overall performance to apply to the opening of hospitals in the United States. In this project, the incidence of deaths within 30 days of 1790 patients undergoing spinal metastasis surgery was 8.49%. The best-performing ML algorithm model developed in this project performed in terms of offset and calibration. With the continuous growth of tumor data, this system can significantly enhance the hospital’s prediction and management of patients with spinal tumors.

### 6.4. Spinal Cord Compression

One of the important pathogenic factors of spinal diseases is the compression of the spinal cord by the bone structure, so research on spinal cord imaging has always been a hot field of AI. In 2017, Gros et al. [76] proposed the OptiC algorithm model, which can automatically, quickly, and accurately segment the brain and spine regions in MRI images and can mark the spinal cord centerline. OptiC’s recognition rate of the gold standard centerline is 98.77%, with an average error of 1.02 mm; the recognition accuracy of the brain region is 99%, and the distance error between the brain and spine region is 9.37 mm, which can be used for spinal cord image analysis. In 2018, Pham et al. [77] compared the differences between ML and manual annotation in spinal cord cell count and immunohistochemical image segmentation. They believe that it is impossible to count stained cell nuclei in c-fos protein with traditional manual methods, but ML technologies such as Random Forest (RF) and SVM can be completed quickly and have a high accuracy rate. This technology also helps to strengthen the ability of immunohistochemical analysis of the spinal cord. In 2018, Wang et al. [78] used a 14-layer deep convolutional neural network to identify multiple sclerosis of the spinal cord. The sensitivity of the results was (98.77 ± 0.35)%, and the specificity was (98.76 ± 0.58)%. The accuracy was (98.77 ± 0.39)%, which outperforms traditional CNNs. In 2019, Aoe et al. [79] proposed a new type of deep neural network, M-Net, which can identify and classify various neurological diseases such as myelopathy through magnetoencephalography (MEG) signals. The accuracy of M-Net’s classification of myelopathy for healthy people and patients is (70.7 ± 10.6)%, and the classification specificity of each disease ranges from 86% to 94%.

### 6.5. Cervical Spondylosis

There are many changes in the structure of the cervical vertebral body, but AI research on the cervical spine has gradually increased in recent years. In 2017, Wang et al. [80] proposed an automated framework that combines diffusion tensor imaging (DTI) indicators with ML algorithms to accurately classify control groups and cervical spondylotic myelopathy (CSM). The SVM classifier has an accuracy of 95.73%, a sensitivity of 93.41%, and a specificity of 98.64%. This method can detect the spinal cord lesions in CSM and provide a surgical reference for spine surgeons. In 2018, Arif et al. [81] proposed an automatic segmentation model for cervical spine X-ray images. The framework first uses a convolutional neural network to locate the spine region in the image and then uses probabilistic space regression to locate the center of the vertebral body. The network segments the cones in the image. The model uses 124 X-rays for training and tests on another 172 X-rays. The accuracy is 84%, and the actual error is 1.69 mm.

In 2015, Chang et al. [82] combined ML and finite element analysis to determine the best internal fixation screw direction for anterior cervical discectomy fusion (ACDF), and the most stable direction of nail insertion that they found provides a surgical reference for spine surgeons. In 2018, Arvind et al. [83] tried to use ML to predict postoperative complications of ACDF, using ANN, logistic regression (LR), SVM, and RF models trained in multiple centers. A total of 20,879 patients underwent ACDF surgery. Analysis shows that ANN and LR algorithms are more reasonable. The sensitivity of ANN is higher than that of LR. The training of large datasets and the application of ML models are promising for improvements in risk prediction. In 2019, Karhade et al. [84] used an ML model to predict whether or not opioids will be used after surgery in 2737 ACDF-treated patients. The accuracy of the model was 81%, and it was concluded that 10% of ACDF patients will use opioids after surgery. ML algorithms can be used to analyze the risks of these patients before surgery, and early intervention can be implemented to reduce the possibility that this population takes opioids for an extended period of time.

### 6.6. Osteoporosis

Regarding the crossover study of AI in the field of vertebral osteoporosis, in 2017, Shioji et al. [85] constructed an algorithm model based on the two variables of mineral density and bone loss rate to predict whether postmenopausal Japanese women would have osteoporosis. The average bone loss rate of the lumbar spine and femoral bone density was 69.4% and 60.9%. The statistical model of the ANN is more accurate than multiple regression analysis and provides help for the early diagnosis and intervention of female osteoporosis. In 2018, Muehlematter et al. [86] proposed using texture analysis and ML on standard CT images to detect the risk of mild vertebral fractures. The researchers collected 58 standard CT scan images of patients with spinal insufficiency, using open-source software. The software extracts the TA features of all vertebral bodies and performs risk prediction based on the supervised training ML model, with an accuracy rate of 97%. In 2019, Mehta et al. [87] used (optimal in terms of hyperparameter tuning) RF and SVM classifiers in dual energy X-ray absorptiometry (dual energy X-ray absorptiometry (DEXA)). To detect sporadic osteoblast metastasis in DEXA, the researchers analyzed the data of 200 patients, and 80% of the data was used for training, while 20% of the data was used for verification. The sensitivity, specificity, and accuracy of the test results were, respectively, 77.8%, 100.0%, and 98.0%. The researchers believe that ML can be used as an auxiliary means to identify sporadic lumbar osteoblast metastasis.

## 7. Quantitative Image Analysis

### 7.1. Localization and Labeling of Spinal Structures

Kelm et al. [49] used an iterative MSL algorithm to locate the intervertebral discs in CT and MRI images, respectively. In order to determine the search range of the intervertebral disc, firstly, the given vertebral body is roughly positioned, the MSL algorithm is then used to highlight the position of the intervertebral disc, and a global spine probability model is finally used to match the marked intervertebral disc. The basis for matching is the shape and coordinate value. The experimental data used 42 T1-weighted MR images and 30 CT images, and the results showed that the accuracy of both MR and CT reached 98.5%. Schwarzenberg et al. [88] also came to the same conclusion Alomari et al. [89] used a two-level probability model to realize the automatic positioning and labeling of intervertebral discs. The model combines high and low levels of information and integrates the appearance and shape information of intervertebral discs as well as the relative spatial relationship between them. Using 105 MRI images of normal and deformed lumbar spines to conduct experiments, the model was first trained, and the remaining data was then tested. The accuracy was as high as 91%, which is relatively high. Glocker et al. [90] confronted the difficulty of vertebrae positioning, subject to pathological thorns such as severe scoliosis and sagittal and distorted fixation devices, and acquired an average positioning error of 8.5 mm between 6 and 12 vertebrae. The suggested method is based on a classified RF, which is trained to detect the position of the vertebrae’s centroid, and employs new ways of obtaining suitable training data and eliminating false positive predictions.

Recent research has also utilized ANNs and DL to identify the structure of the spine. Chen et al. employed RF classification, which implements a deep CNN to drive the first coarse localization-hybrid method; this method enables a significant improvement over prior art not based on DL, with an average localization error of 1.6 to 2 mm [91,92] for the centroid of the intervertebral disc.

In fact, cutting-edge methods for identifying and classifying spinal structures have achieved performance levels on par with those of human experts in the field. Commercial Image Archiving and Communication Systems and commercially accessible clinical imaging software increasingly include detection and labeling features, though the underlying technical specifics are not publicly disclosed.

### 7.2. Segmentation

In terms of spinal image segmentation, in view of the existing characteristics of spinal images, researchers have proposed many algorithms to achieve spinal image segmentation. For example, Ma et al. [93] used the mean information of the spine shape generated by the statistical shape model method to achieve semi-automatic segmentation. In the segmentation process, the position of the spine in the image is first manually determined, and a variety of prior information, such as the shape and gradient of the model, which is used as constraints to achieve segmentation, is then introduced. Li et al. [94] proposed an improved level set (LSM) segmentation method. In actual implementation, in order to solve the problem that the level set function (LSF) is sensitive to image noise, the effect of segmentation on the irregular border of the spine is not effective. The best solution to the problem is to use the gradient information in the image to evolve the LSF to further improve the accuracy. Lim et al. [95] introduced the statistical shape of the spinal image as a priori information to initialize LSF, but this method, though segmentation accuracy was improved, increased the computational complexity of the LSM.

In recent years, with the advent of big data, researchers in the field of DL have developed deep CNNs with multiple hidden layers and complex structures that have powerful feature extraction and feature expression capabilities, enabling DL-related algorithms to make remarkable strides in the field of computer vision, particularly in image recognition, classification, and semantic segmentation. Lessmann et al. [96] developed a 3D CNN with a memory component to remember previously categorized vertebrae. To be able to process massive datasets, the technique employs a 3D sliding window approach that first determines the position at which the window contains a whole vertebra and then applies a deep classifier to perform pixel-level segmentation. The memory is then updated so that, if a segment of previously segmented vertebrae is found while searching for the subsequent vertebrae, it will be disregarded. With an average DSC of 0.94 and an MSD of 0.2 mm, this approach enabled the attainment of exceptional precision.

Haq et al. [97] proposed a 3D segmentation method of MRI images for health and intervertebral disc herniation. It uses a single mesh deformation model to add shape prior information. First, an elliptical mesh is initialized on the edge of the original image of the intervertebral disc. The grid model is then deformed according to the gradient force of the image to finally obtain the true boundary of the intervertebral disc. The test results show that this method can accurately segment healthy and herniated spines. The disadvantage is that, during the deformation process, manual intervention is required to change the single prior shape model for different images.

Neubert [98] proposed a 3D segmentation method based on the registration of the statistical shape model and the gray-level intensity distribution. Compared with the manual segmentation results, the processing results have very small errors, and the accuracy reaches 98.3%.

The labeling and segmentation of spinal images is the basis and key to the development of auxiliary diagnosis and treatment, and it is also a relatively mature field. In 2017, Forsberg et al. [99] used MRI images of the vertebral body manually annotated by clinicians to train a DL model for vertebral body recognition. The detection sensitivity, accuracy, and accuracy rate were 99.1–99.8%, 99.6–100% and 98.8–99.8%, respectively. The results show that it is feasible to use DL technology to assist radiologists in the rapid identification of vertebral bodies. In 2017, Belharbi et al. [100] used the convolutional network model to realize the positioning of the L3 vertebral body on the axial CT through transfer learning, which plays an important role in the positioning of the entire lumbar spine. The advantage is that, after the model is pre-trained by the ImageNet database, it does not require a large amount of expert annotation data to complete the training. The researchers tested 642 CT scans from different patients. The average positioning error was 1.91 ± 2.69 frames (<5 mm), and the accuracy reached the requirements of routine clinical examinations. In 2018, Galbusera et al. [101] used generative adversarial networks to realize the mapping and conversion between X-ray and MRI’s T1W image, T2W image, STIR image, and TIRM image, showing high consistency (Κ = 0.691), which shows that the conditional generation adversarial network can complete the convincing ultra-high resolution spinal image conversion task. Gawel et al. [102] introduced a new method of segmenting vertebral bodies. This method combines a variety of ML techniques, uses a cascade classifier for automatic vertebral body recognition, and then uses the active appearance model to segment again. The results show that this model has correct algorithm convergence, and the accuracy is relatively high [FF = (90.19 ± 1.01)%]. In 2019, Lessmann et al. [103] proposed an iterative neural network model that automatically recognizes and classifies vertebrae. The model is evaluated based on CT and MRI, covering different segments of vertebrae, and is anatomically accurate. Compared with the prior art method, this iterative segmentation method is faster, more flexible, and versatile.

### 7.3. Outcome Prediction

Since its inception, the healthcare industry has exhibited an interest in predictive analytics due to its vast potential for enhancing patient care and financial administration. The healthcare applications of predictive analytics include the identification of chronic patients at risk for a poor health outcome and who may benefit from interventions, the development of personalized medicine and therapies, the prediction of adverse events during hospitalization, and the optimization of the supply chain.

In the past 10 years, a number of studies have provided models for predicting various aspects of the outcome of spinal surgery; a sample of these models is outlined here. McGirt et al. [104] utilized simple approaches drawn from statistics, such as linear and logistic regression, to predict values such as the Oswestry Disability Index (ODI) [105] one year after surgery, the occurrence of complications, readmission to the hospital, and return to work. The accuracy of the prediction model for complications and return to work ranged from 72% to 84%, based on data from 750 to 1200 patients. The model considered more than 40 predictors, including the preoperative ODI, age, ethnicity, body mass index, a full description of the symptoms, the likely presence of additional spinal illnesses, and a number of ratings describing the patient’s health and functional state. Relatively recently, Kim et al. [60] used logistic regression and a shallow ANN to specifically predict the occurrence of four types of major complications in patients undergoing spine fusion, namely, cardiac complications, wound complications, venous thromboembolism, and mortality, and achieved significantly better results than when employing the clinical score typically employed for such applications. Lee et al. [106] utilized a similar approach to predict surgical site infections. Intriguingly, a subsequent study undertook an external validation of this predictive model based on another patient sample, revealing a number of flaws and demonstrating a generally poor performance [107]. Recent research [108] used an ensemble of decision trees to predict, with an overall accuracy of 87.6%, severe intraoperative or perioperative problems following adult spine deformity surgery. Durand et al. examined a different outcome, i.e., the need for blood transfusion following adult deformity surgery, which was accurately predicted using single decision trees and random forests [109].

## 8. Future Applications

The integration of AI into biomechanical investigations is an additional frontier in spinal surgery research. In this industry, AI has promising applications, despite the fact that its use is still in its infancy. The analysis of gait and motion patterns, as well as the identification of abnormal gait in spinal illnesses, is one area that can benefit from the application of AI [110,111,112]. For example, AI and ML have had less impact on fundamental biomechanics compared to clinical and radiological applications. In recent years, however, a few articles documenting the use of ANNs for classic biomechanical problems, such as the calculation of loads and stresses, have begun to appear. Despite the fact that there are no available studies on spine biomechanics, we believe that it is worthwhile to briefly mention some ML-based studies investigating other musculoskeletal districts, as a review of the current state of the art may aid in defining potential future applications of ML techniques in spine biomechanics.

Zadpoor et al. [113] explored a comparable issue: the prediction of the mechanical loads that determine particular mechanical properties of a biological tissue undergoing remodeling, namely, trabecular bone. Using an existing biomechanical computer model capable of predicting bone tissue adaptation under mechanical loading based on local strains, the scientists ran a series of simulations in which random loads were given to a small sample of bone trabecular tissue. The outputs of the simulations, i.e., the reconstructed local bone densities, were utilized to train an ANN to predict the loads that caused this type of remodeling. An additional application of AI is the calculation of stresses in patient-specific analyses, hence reducing the need for computationally expensive finite element models. For instance, Lu et al. constructed a shallow ANN capable of predicting the stress in the cartilage of the knee joint’s tibial plateau and femoral condyles. A finite element model of the knee was used to generate a dataset that was then used to train an ANN, which was able to predict the stress in each element of the articular cartilage with a significant reduction in time and cost compared to creating and solving the finite element model itself [114].

The use of AI approaches in musculoskeletal biomechanics appears to be in its infancy; the few published articles have not yet exploited the promise of the most recent advances, such as DL. Despite this, the existing literature demonstrates AI’s potential in this subject. This method would promote the general use of patient-specific modeling in bench-to-bedside applications, where the computational resources and time necessary for the design and solution of a traditional biomechanical model may clash with clinical requirements.

## 9. Discussion

According to the aforementioned study states, deep learning is performing at the highest level, even though artificial intelligence in spinal imaging has been widely used and improved quickly. However, there are still many issues in this area that require further research. The use of connected spinal scans must win the complete confidence of the medical community, and more work needs to be put into the creation of artificial intelligence. For this area, we provide a few examples.

First, spinal image analysis will be more heavily influenced by AI. Every day, numerous photos are produced by international medical institutes; nevertheless, manual annotation by professionals is a time-consuming and labor-intensive operation [115]. The continuously expanding volume of data is still challenging to manage, despite the fact that there are currently graphical interface tools to facilitate annotation.

Second, the development and enhancement of standardized picture datasets will also be a key area of focus. There are now only a few platforms for spinal image data, many of the data are private, and the sample size of the data is very small [116]. A pretreatment procedure must be employed to balance the discrepancies between photos when compiling a big dataset [117]. A labeling platform needs to be established or improved to enable experts to label in accordance with common standards.

Third, one of the main areas of research in this area should be the collaborative application of multimodal data [118]. A single kind of image is extremely specialized and exclusively performs particular imaging tasks. Therefore, clinical or experimental data, such as information on clinical diagnosis and therapy in electronic medical records, should be used extensively [119]. To extract them, employing text recognition and natural language processing techniques needs to be considered. It is also a crucial stage in the creation of standard datasets.

Fourth, there is also a need to address how prediction models can be easier to read. Despite having strong discrimination, artificial intelligence has been criticized for having poor interpretability. On the question of whether interpretability is required in ML, there have been discussions [120]. The interpretability of pictures of the spine should be improved, although some experts feel that model performance is far more important than interpretability.

Fifth, the development of deep learning models will increasingly focus on the processing and interpretation of 3D images of the spine. Biological research and medical diagnostics are increasingly using 3D stereoscopic photographs of bones to retain more detailed and structural information. This can substantially enhance specialists’ interpretation of images. Certain finite element explorations have been used to create 3D models [121,122]. However, the majority of the most advanced artificial intelligence models currently in use, particularly deep learning models, are created for 2D images. Voxels in 3D images must be processed, which means that, in addition to the model’s input structure changing, exponential growth must also be taken into account [123,124]. The difficulties resulting from the massive amount of computation will hinder the advancement of artificial intelligence [125,126].

Sixth, regarding prediction reliability and uncertainty quantification, the current applications of ML in the spinal imaging field (nay, in the entire biomedical informatics field) generally seem to focus on the prediction task, and the model performance is evaluated with such metrics as accuracy, ROC, and sensitivity [127,128,129,130]. However, the much lower attended reliability and uncertainty of the predictions can be of great significance in medical diagnosis [131]. Unreliable predictions can place substantial financial and psychological burdens on the patients’ families. Therefore, the reliability quantification approaches (such as conformal prediction) and how users should weigh and interpret the uncertainties in this field are worthy of study [132].

Finally, data and model communication across different institutes is due to the different patient information protection protocols. Model sharing strategies, rather than data sharing strategies, such as federated learning, merit further study [133].

## 10. Conclusions

Every aspect of the imaging value chain can be significantly improved by AI. By enhancing image quality, patient centricity, imaging efficiency, and diagnostic accuracy, AI can increase the value of spinal images delivered to their patients and referring clinicians. This includes assessing the appropriateness of imaging orders and predicting which patients are at risk for fracture. Emerging, non-disruptive technology has attained a significant level of development, allowing it to have a practical impact on a number of study topics. The fields of computer vision and image processing are gaining traction due to recent advancements in DL and the increased availability of computational resources, such as powerful GPUs. In fact, the majority of recent spinal research projects employing AI are related to medical imaging, but an increasing influence on other domains, such as spine biomechanics, is anticipated in the near future. However, there are still many issues in this area that require more research. Researchers still need to put more work into developing pertinent prediction models if they seek the support of the medical community.

## Data Availability

The study did not report any data.

## Abbreviations

CNN	Convolutional Neural Network
RNN	Recurrent Neural Network
GAN	Generative Adversarial Networks
CT	Computed Tomography
AI	Artificial Intelligence
DL	Deep Learning
ML	Machine Learning
MRI	Magnetic Resonance Imaging
MR	Magnetic resonance
CS	Compressed sensing
PACS	Picture Archiving & Communication System
MSL	Marginal space learning
FAST	Fully assisting scanner technologies
PSNR	Peak signal-to-noise ratio
MTL	Multi-task learning

## References

[1] Harkey P., Duszak R.J., Gyftopoulos S., Rosenkrantz A.B. (2018). Who refers musculoskeletal extremity imaging examinations to radiologists?. AJR.

[2] Doshi A.M., Moore W.H., Kim D.C., Rosenkrantz A.B., Fefferman N.R., Ostrow D.L., Recht M.P. (2018). Informatics solutions for driving an effective and efficient radiology practice. RadioGraphics.

[3] Nam K.H., Kim D.H., Choi B.K., Han I.H. (2019). Internet of Things, Digital Biomarker, and Artificial Intelligence in Spine: Current and Future Perspectives. Neurospine.

[4] Kim Y.J., Ganbold B., Kim K.G. (2020). Web-Based Spine Segmentation Using Deep Learning in Computed Tomography Images. Healthc. Inform. Res..

[5] Rasouli J.J., Shao J., Neifert S., Gibbs W.N., Habboub G., Steinmetz M.P., Mroz T.E. (2020). Artificial Intelligence and Robotics in Spine Surgery. Glob. Spine J..

[6] Hosny A., Parmar C., Quackenbush J., Schwartz L.H., Aerts H.J. (2018). Artificial intelligence in radiology. Nat. Rev. Cancer.

[7] Galbusera F., Casaroli G., Bassani T. (2019). Artificial intelligence and machine learning in spine research. JOR Spine.

[8] Bertsimas D., Masiakos P.T., Mylonas K.S., Wiberg H. (2019). Prediction of Cervical Spine Injury in Young Pediatric Patients: An Optimal Trees Artificial Intelligence Approach. J. Pediatric Surg..

[9] Poole D.L., Mackworth A.K., Goebel R. (1998). Computational intelligence and knowledge. Computational Intelligence: A Logical Approach.

[10] LeCun Y., Bengio Y., Hinton G. (2015). Deep learning. Nature.

[11] Krizhevsky A., Sutskever I., Hinton G.E. (2012). ImageNet classification with deep convolutional neural networks. Proceedings of the 25th International Conference on Neural Information Processing Systems.

[12] Chen L.C., Papandreou G., Kokkinos I., Murphy K., Yullie A.L. (2018). DeepLab: Semantic image segmentation with deep convolutional nets, atrous convolution, and fully connected CRFs. IEEE Trans. Pattern. Anal. Mach. Intell..

[13] Batista-García-Ramó K., Fernández-Verdecia C.I. (2018). What we know about the brain structure–function relationship. Behav. Sci..

[14] Sun T. (2019). Applying deep learning to audit procedures: An illustrative framework. Account. Horiz..

[15] Jarrahi M.H. (2018). Artificial intelligence and the future of work: Human-AI symbiosis in organizational decision making. Bus. Horiz..

[16] Tussyadiah I. (2020). A review of research into automation in tourism: Launching the Annals of Tourism Research Curated Collection on Artificial Intelligence and Robotics in Tourism. Ann. Tour. Res..

[17] Chuah SH W., Yu J. (2021). The future of service: The power of emotion in human-robot interaction. J. Retail. Consum. Serv..

[18] Chai X. (2020). Diagnosis method of thyroid disease combining knowledge graph and deep learning. IEEE Access.

[19] Tang J., Liu G., Pan Q. (2021). A review on representative swarm intelligence algorithms for solving optimization problems: Applications and trends. IEEE/CAA J. Autom. Sin..

[20] Gaeta M., Loia F., Sarno D., Carrubbo L. (2019). Online social network viability: Misinformation management based on service and systems theories. Int. J. Bus. Manag..

[21] Wang J., Yang Y., Wang T., Sherratt R.S., Zhang J. (2020). Big data service architecture: A survey. J. Internet Technol..

[22] Duan M., Li K., Liao X., Li K. (2017). A parallel multiclassification algorithm for big data using an extreme learning machine. IEEE Trans. Neural Netw. Learn. Syst..

[23] Hoang D.T., Kang H.J. (2019). Rolling element bearing fault diagnosis using convolutional neural network and vibration image. Cogn. Syst. Res..

[24] Moshayedi A.J., Roy A.S., Kolahdooz A., Shuxin Y. (2022). Deep learning application pros and cons over algorithm. EAI Endorsed Trans. AI Robot..

[25] Luo Y., Tseng H.H., Cui S., Wei L., Ten Haken R.K., El Naqa I. (2019). Balancing accuracy and interpretability of machine learning approaches for radiation treatment outcomes modeling. BJR Open.

[26] Huang J., Shen H., Wu J., Hu X., Zhu Z., Lv X., Liu Y., Wang Y. (2020). Spine Explorer: A deep learning based fully automated program for efficient and reliable quantifications of the vertebrae and discs on sagittal lumbar spine MR images. Spine J..

[27] Pfirrmann C.W., Metzdorf A., Zanetti M., Hodler J., Boos N. (2001). Magnetic resonance classification of lumbar intervertebral disc degeneration. Spine.

[28] Szegedy C., Ioffe S., Vanhoucke V., Alemi A.A. Inception-v4, inception-resnet and the impact of residual connections on learning. Proceedings of the Thirty-First AAAI Conference on Artificial Intelligence.

[29] Ronneberger O., Fischer P., Brox T. (2015). U-net: Convolutional networks for biomedical image segmentation. Proceedings of the International Conference on Medical Image Computing and Computer-Assisted Intervention.

[30] Teede H.J., Misso M.L., Boyle J.A., Garad R.M., McAllister V., Downes L., Woolcock J. (2018). Translation and implementation of the Australian-led PCOS guideline: Clinical summary and translation resources from the International Evidence-based Guideline for the Assessment and management of polycystic ovary syndrome. Med. J. Aust..

[31] Lee S., Choe E.K., Kang H.Y., Yoon J.W., Kim H.S. (2020). The exploration of feature extraction and machine learning for predicting bone density from simple spine X-ray images in a Korean population. Skelet. Radiol..

[32] Fischler M.A. (1983). Image Understanding Research and Its Application to Cartography and Computer-Based Analysis of Aerial Imagery.

[33] Lakhani P., Prater A.B., Hutson R.K., Andriole K.P., Dreyer K.J., Morey J., Hawkins C.M. (2018). Machine learning in radiology: Applications beyond image interpretation. J. Am. Coll. Radiol..

[34] Lee Y.H. (2018). Efficiency improvement in a busy radiology practice: Determination of musculoskeletal magnetic resonance imaging protocol using deep-learning convolutional neural networks. J. Digit. Imaging.

[35] Trivedi H., Mesterhazy J., Laguna B., Vu T., Sohn J.H. (2018). Automatic determination of the need for intravenous contrast in musculoskeletal MRI examinations using IBM Watson’s natural language processing algorithm. J. Digit. Imaging.

[36] Kohli M., Dreyer K.J., Geis J.R. (2015). Rethinking radiology informatics. AJR.

[37] Mardani M., Gong E., Cheng J.Y., Vasanawala S.S., Zaharchuk G., Xing L., Pauly J.M. (2019). Deep Generative Adversarial Neural Networks for Compressive Sensing MRI. IEEE Trans. Med. Imaging.

[38] Goodfellow I., Pouget-Abadie J., Mirza M., Xu B., Warde-Farley D., Ozair S., Bengio Y. (2020). Generative adversarial networks. Commun. ACM.

[39] Schlemper J., Caballero J., Hajnal J.V., Price A., Rueckert D. (2018). A Deep Cascade of Convolutional Neural Networks for Dynamic MR Image Reconstruction. IEEE Trans. Med. Imaging.

[40] Chen M., Wang Y., Sun A. (2018). Advantages of joint multi-echo MRI reconstruction via deep learning. Proceedings of the ISMRM Workshop on Machine Learning Part II.

[41] Oktay O., Schlemper J., Folgoc L.L., Lee M., Heinrich M., Misawa K., Rueckert D. (2018). Attention u-net: Learning where to look for the pancreas. arXiv.

[42] Eo T., Jun Y., Kim T., Jang J., Lee H.J., Hwang D. (2018). KIKI-net: Cross-domain convolutional neural networks for reconstructing undersampled magnetic resonance images. Magn. Reson. Med..

[43] Subhas N., Pursyko C.P., Polster J.M., Obuchowski N.A., Primak A.N., Dong F.F., Herts B.R. (2018). Dose reduction with dedicated CT metal artifact reduction algorithm: CT phantom study. AJR.

[44] Subhas N., Polster J.M., Obuchowski N.A., Primak A.N., Dong F.F., Herts B.R., Iannotti J.P. (2016). Imaging of arthroplasties: Improved image quality and lesion detection with iterative metal artifact reduction, a new CT metal artifact reduction technique. AJR.

[45] Wolterink J.M., Leiner T., Viergever M.A., Išgum I. (2017). Generative Adversarial Networks for Noise Reduction in Low-Dose CT. IEEE Trans. Med. Imaging.

[46] Giordano C., Monica I., Quattrini F., Villaggi E., Gobbi R., Barbattini L. (2020). Evaluation of the radiation dose to the hands of orthopaedic surgeons during fluoroscopy using stored images. Radiat. Prot. Dosim..

[47] McDonald R.J., Schwartz K.M., Eckel L.J., Diehn F.E., Hunt C.H., Bartholmai B.J., Kallmes D.F. (2015). The effects of changes in utilization and technological advancements of cross-sectional imaging on radiologist workload. Acad. Radiol..

[48] Wang T., Iankoulski A. (2019). Intelligent tools for a productive radiologist workflow: How machine learning enriches hanging protocols. GE Healthc. Website.

[49] Kelm B.M., Wels M., Zhou S.K., Seifert S., Suehling M., Zheng Y., Comaniciu D. (2013). Spine detection in CT and MR using iterated marginal space learning. Med. Image Anal..

[50] Ghesu F.C., Georgescu B., Mansi T., Neumann D., Hornegger J., Comaniciu D. (2016). An artificial agent for anatomical landmark detection in medical images. Medical Image Computing and Computer-Assisted Intervention—MICCAI 2016.

[51] Zhang P., Zheng Y. (2019). Unsupervised Deep Representation Learning for Fine-grained Body Part Recognition. U.S. Patent.

[52] Chen H., Zhang Y., Kalra M.K., Lin F., Chen Y., Liao P., Wang G. (2017). Low-Dose CT with a Residual Encoder-Decoder Convolutional Neural Network (RED-CNN). IEEE Trans. Med. Imaging.

[53] Li Z., Zhou S., Huang J., Yu L., Jin M. (2020). Investigation of low-dose CT image denoising using unpaired deep learning methods. IEEE Trans. Radiat. Plasma Med. Sci..

[54] Han Y., Ye J.C. (2018). Framing U-Net via Deep Convolutional Framelets: Application to Sparse-View CT. IEEE Trans. Med. Imaging.

[55] Anirudh R., Kim H., Thiagarajan J.J., Mohan K.A., Champley K., Bremer T. Lose the Views: Limited Angle CT Reconstruction via Implicit Sinogram Completion. Proceedings of the IEEE Conference on Computer Vision and Pattern Recognition (CVPR).

[56] Xu J., Gong E., Pauly J., Zaharchuk G. (2017). 200× low-dose PET reconstruction using deep learning. Proceedings of the NIPS Workshop on Machine Learning for Health.

[57] Han L., Ju G., Mei L. (2018). Multitask DNN for liver imaging enhancement. Proceedings of the ISMRM Workshop on Machine Learning Part II.

[58] Azimi P., Mohammadi H.R., Benzel E.C., Shahzadi S., Azhari S. (2017). Use of artificial neural networks to decision making in patients with lumbar spinal canal stenosis. J. Neurosurg. Sci..

[59] Han Z., Wei B., Leung S., Nachum I.B., Laidley D., Li S. (2018). Automated pathogenesis-based diagnosis of lumbar neural foraminal stenosis via deep multiscale multitask learning. Neuroinformatics.

[60] Kim J.S., Merrill R.K., Arvind V., Kaji D., Pasik S.D., Nwachukwu C.C., Cho S.K. (2018). Examining the ability of artificial neural networks machine learning models to accurately predict complications following posterior lumbar spine fusion. Spine.

[61] Staartjes V.E., Marlies P., Vandertop W.P., Schröder M.L. (2019). Deep learning-based preoperative predictive analytics for patient-reported outcomes following lumbar discectomy: Feasibility of center specific modeling. Spine J..

[62] Ramirez L., Durdle N.G., Raso V.J., Hill D.L. (2006). A support vector machines classifier to assess the severity of idiopathic scoliosis from surface topography. IEEE Trans. Inf. Technol. Biomed..

[63] Bergeron C., Cheriet F., Ronsky J., Zernicke R., Labelle H. (2005). Prediction of anterior scoliotic spinal curve from trunk surface using support vector regression. Eng. Appl. Artif. Intel..

[64] Lenke L.G., Edwards C.C., Bridwell K.H. (2003). The Lenke classification of adolescent idiopathic scoliosis: How it organizes curve patterns as a template to perform selective fusions of the spine. Spine.

[65] Seoud L., Adankon M.M., Labelle H., Dansereau J., Cheriet F. (2010). Prediction of scoliosis curve type based on the analysis of trunk surface topography. Proceedings of the 2010 IEEE International Symposium on Biomedical Imaging: From Nano to Macro.

[66] Komeili A., Westover L., Parent E.C., El-Rich M., Adeeb S. (2015). Monitoring for idiopathic scoliosis curve progression using surface topography asymmetry analysis of the torso in adolescents. Spine J..

[67] Zhang J., Lou E., Le L.H., Hill D.L., Raso J.V., Wang Y. (2009). Automatic Cobb measurement of scoliosis based on fuzzy Hough transform with vertebral shape prior. J. Digit. Imaging.

[68] Galbusera F., Niemeyer F., Wilke H.J., Bassani T., Casaroli G., Anania C., Sconfienza L.M. (2019). Fully automated radiological analysis of spinal disorders and deformities: A deep learning approach. Eur. Spine J..

[69] Zhang J., Li H., Lv L., Zhang Y. (2017). Computer-aided cobb measurement based on automatic detection of vertebral slopes using deep neural network. Int. J. Biomed. Imaging.

[70] Wu H., Bailey C., Rasoulinejad P., Li S. (2018). Automated comprehensive Adolescent Idiopathic Scoliosis assessment using MVC-Net. Med. Image Anal..

[71] Thong W.E., Parent S., Wu J., Aubin C.E., Labelle H., Kadoury S. (2016). Three-dimensional morphology study of surgical adolescent idiopathic scoliosis patient from encoded geometric models. Eur. Spine J..

[72] Bi W.L., Hosny A., Schabath M.B., Giger M.L., Birkbak N.J., Mehrtash A., Aerts H.J. (2019). Artificial intelligence in cancer imaging: Clinical challenges and applications. CA Cancer J. Clin..

[73] Wang J., Fang Z., Lang N., Yuan H., Su M.Y., Baldi P. (2017). A multi-resolution approach for spinal metastasis detection using deep Siamese neural networks. Comput. Biol. Med..

[74] Lang N., Zhang Y., Zhang E., Zhang J., Chow D., Chang P., Su M.Y. (2019). Differentiation of spinal metastases originated from lung and other cancers using radiomics and deep learning based on DCE-MRI. Magn. Reson. Imaging.

[75] Karhade A.V., Thio Q.C., Ogink P.T., Shah A.A., Bono C.M., Oh K.S., Schwab J.H. (2019). Development of machine learning algorithms for prediction of 30-day mortality after surgery for spinal metastasis. Neurosurgery.

[76] Gros C., De Leener B., Dupont S.M., Martin A.R., Fehlings M.G., Bakshi R., Sdika M. (2018). Automatic spinal cord localization, robust to MRI contrasts using global curve optimization. Med. Image Anal..

[77] Pham B., Gaonkar B., Whitehead W., Moran S., Dai Q., Macyszyn L., Edgerton V.R. (2018). Cell counting and segmentation of immunohistochemical images in the spinal cord: Comparing deep learning and traditional approaches. Conf. Proc. IEEE Eng. Med. Biol. Soc..

[78] Wang S.H., Tang C., Sun J., Yang J., Huang C., Phillips P., Zhang Y.D. (2018). Multiple sclerosis identifica-tion by 14-layer convolutional neural network with batch normalization, dropout, and stochastic pooling. Front. Neurosci..

[79] Aoe J., Fukuma R., Yanagisawa T., Harada T., Tanaka M., Kobayashi M., Kishima H. (2019). Automatic diagnosis of neurological diseases using MEG signals with a deep neural network. Sci. Rep..

[80] Wang S., Hu Y., Shen Y., Li H. (2018). Classification of diffusion tensor metrics for the diagnosis of a myelopathic cord using machine learning. Int. J. Neural Syst..

[81] Al Arif S., Knapp K., Slabaugh G. (2018). Fully automatic cervical vertebrae segmentation framework for X-ray images. Comput. Methods Programs Biomed..

[82] Chang T.K., Hsu C.C., Chen K.T. (2015). Optimal screw orientation for the fixation of cervical degenerative disc disease using nonlinear C3-T2 multi-level spinal models and neuro-genetic algorithms. Acta Bioeng. Biomech..

[83] Arvind V., Kim J.S., Oermann E.K., Kaji D., Cho S.K. (2018). Predicting Surgical Complications in Adult Patients Undergoing Anterior Cervical Discectomy and Fusion Using Machine Learning. Neurospine.

[84] Karhade A.V., Ogink P.T., Thio Q.C., Broekman M.L., Cha T.D., Hershman S.H., Schwab J.H. (2019). Machine learning for prediction of sustained opioid prescription after anterior cervical discectomy and fusion. Spine J..

[85] Shioji M., Yamamoto T., Ibata T., Tsuda T., Adachi K., Yoshimura N. (2017). Artificial neural networks to predict future bone mineral density and bone loss rate in Japanese postmenopausal women. BMC Res. Notes.

[86] Muehlematter U.J., Mannil M., Becker A.S., Vokinger K.N., Finkenstaedt T., Osterhoff G., Guggenberger R. (2019). Vertebral body insufficiency fractures: Detection of vertebrae at risk on standard CT images using texture analysis and machine learning. Eur. Radiol..

[87] Mehta S.D., Sebro R. (2019). Random forest classifiers aid in the detection of incidental osteoblastic osseous metastases in DEXA studies. Int. J. Comput. Assist. Radiol. Surg..

[88] Schwarzenberg R., Freisleben B., Nimsky C., Egger J. (2014). Cube-cut: Vertebral body segmentation in MRI-data through cubic-shaped divergences. PLoS ONE.

[89] Alomari R.S., Corso J.J., Chaudhary V. (2011). Labeling of Lumbar Discs Using Both Pixel- and Object-Level Features with a Two-Level Probabilistic Model. IEEE Trans. Med. Imaging.

[90] Glocker B., Feulner J., Criminisi A., Haynor D.R., Konukoglu E. (2012). Automatic Localization and Identification of Vertebrae in Arbitrary Field-of-View CT Scans. Medical Image Computing and Computer-Assisted Intervention—MICCAI 2012.

[91] Chen C., Belavy D., Yu W., Chu C., Armbrecht G., Bansmann M., Zheng G. (2015). Localization and segmentation of 3D intervertebral discs in MR images by data driven estimation. IEEE Trans. Med. Imaging.

[92] Chen H., Shen C., Qin J., Ni D., Shi L., Cheng J.C., Heng P.A. (2015). Automatic Localization and Identification of Vertebrae in Spine CT via a Joint Learning Model with Deep Neural Networks. Medical Image Computing and Computer-Assisted Intervention—MICCAI 2015.

[93] Ma J., Lu L. (2013). Hierarchical segmentation and identification of thoracic vertebra using learning based edge detection and coarse-to-fine deformable model. Comput. Vis. Image Underst..

[94] Li C., Xu C., Gui C. (2010). Distance regularized level set evolution and its application to image segmentation. IEEE Trans. Image Process..

[95] Lim P.H., Bagci U., Bai L. (2013). Introducing Wilmore flow into level set segmentation of spinal vertebrae. IEEE Trans. Biomed. Eng..

[96] Lessmann N., van Ginneken B., Išgum I. (2018). Iterative convolutional neural networks for automatic vertebra identification and segmentation in CT images. arXiv.

[97] Haq R., Aras R., Besachio D.A., Borgie R.C., Audette M.A. (2015). 3D lumbar spine intervertebral disc segmentation and compression simulation from MRI using shape-aware models. Int. J. Comput. Assist. Radiol. Surg..

[98] Neubert A., Fripp J., Engstrom C., Schwarz R., Lauer L., Salvado O., Crozier S. (2012). Automated detection, 3D segmentation and analysis of high resolution spine MR images using statistical shape models. Phys. Med. Biol..

[99] Forsberg D., Sjoblom E., Sunshine J.L. (2017). Detection and labeling of vertebrae in MR images using deep learning with clinical annotations as training data. J. Digit. Imaging.

[100] Belharbi S., Chatelain C., Hérault R., Adam S., Thureau S., Chastan M., Modzelewski R. (2017). Spotting L3 slice in CT scans using deep convolutional network and transfer learning. Comput. Biol. Med..

[101] Galbusera F., Bassani T., Casaroli G., Gitto S., Zanchetta E., Costa F., Sconfienza L.M. (2018). Generative models: An upcoming innovation in musculoskeletal radiology? A pre-liminary test in spine imaging. Eur. Radiol. Exp..

[102] Gaweł D., Główka P., Kotwicki T., Nowak M. (2018). Automatic spine tissue segmentation from mri data based on cascade of boosted classifiers and active appearance model. Biomed. Res. Int..

[103] Lessmann N., Van Ginneken B., De Jong P.A., Išgum I. (2019). Iterative fully convolutional neural networks for automatic vertebra segmentation and identification. Med. Image Anal..

[104] McGirt M.J., Sivaganesan A., Asher A.L., Devin C.J. (2015). Prediction model for outcome after low-back surgery: Individualized likelihood of complication, hospital readmission, return to work, and 12-month improvement in functional disability. Neurosurg Focus..

[105] Fairbank J.C., Couper J., Davies J.B., O’Brien J.P. (1980). The Oswestry low back pain disability questionnaire. Physiotherapy.

[106] Lee M.J., Cizik A.M., Hamilton D., Chapman J.R. (2014). Predicting surgical site infection after spine surgery: A validated model using a prospective surgical registry. Spine J..

[107] Janssen D.M., van Kuijk S.M., d’Aumerie B.B., Willems P.C. (2018). External validation of a prediction model for surgical site infection after thoracolumbar spine surgery in a Western European cohort. J. Orthop. Surg. Res..

[108] Scheer J.K., Smith J.S., Schwab F., Lafage V., Shaffrey C.I., Bess S., Ames C.P. (2017). Development of a preoperative predictive model for major complications following adult spinal deformity surgery. J. Neurosurg. Spine.

[109] Durand W.M., DePasse J.M., Daniels A.H. (2018). Predictive modeling for blood transfusion after adult spinal deformity surgery: A tree-based machine learning approach. Spine.

[110] Fukuchi R.K., Eskofier B.M., Duarte M., Ferber R. (2011). Support vector machines for detecting age-related changes in running kinematics. J. Biomech..

[111] Leardini A., Biagi F., Merlo A., Belvedere C., Benedetti M.G. (2011). Multi-segment trunk kinematics during locomotion and elementary exercises. Clin. Biomech..

[112] Zhang J., Lockhart T.E., Soangra R. (2014). Classifying lower extremity muscle fatigue during walking using machine learning and inertial sensors. Ann. Biomed. Eng..

[113] Zadpoor A.A., Campoli G., Weinans H. (2013). Neural network prediction of load from the morphology of trabecular bone. App. Math. Model..

[114] Lu Y., Pulasani P.R., Derakhshani R., Guess T.M. (2013). Application of neural networks for the prediction of cartilage stress in a musculoskeletal system. Biomed. Signal. Process. Control..

[115] Sun X., Xv H., Dong J., Zhou H., Chen C., Li Q. (2020). Few-shot learning for domain-specific fine-grained image classification. IEEE Trans. Ind. Electron..

[116] Goldman M.J., Craft B., Hastie M., Repečka K., McDade F., Kamath A., Haussler D. (2020). Visualizing and interpreting cancer genomics data via the Xena platform. Nat. Biotechnol..

[117] Zhou L., Zhang C., Liu F., Qiu Z., He Y. (2019). Application of deep learning in food: A review. Compr. Rev. Food Sci. Food Saf..

[118] Sharma K., Giannakos M. (2020). Multimodal data capabilities for learning: What can multimodal data tell us about learning. Br. J. Educ. Technol..

[119] Ruan T., Lei L., Zhou Y., Zhai J., Zhang L., He P., Gao J. (2019). Representation learning for clinical time series prediction tasks in electronic health records. BMC Med. Inform. Decis. Mak..

[120] Krishnan M. (2020). Against interpretability: A critical examination of the interpretability problem in machine learning. Philos. Technol..

[121] Cui Y., Shen H., Chen Y., Zhang W., Zhu J., Duan Z., Weiqiang L. (2022). Study on the process of intervertebral disc disease by the theory of continuum damage mechanics. Clin. Biomech..

[122] Cui Y., Xiang D., Shu L., Duan Z., Liao Z., Wang S., Liu W. (2022). Incremental element deletion-based finite element analysis of the effects of impact speeds, fall postures, and cortical thicknesses on femur fracture. Materials.

[123] Ji M., Gall J., Zheng H., Liu Y., Fang L. Surfacenet: An end-to-end 3d neural network for multiview stereopsis. Proceedings of the IEEE International Conference on Computer Vision.

[124] Bebeshko B., Khorolska K., Kotenko N., Desiatko A., Sauanova K., Sagyndykova S., Tyshchenko D. (2021). 3D modelling by means of artificial intelligence. J. Theor. Appl. Inf. Technol..

[125] Grace K., Salvatier J., Dafoe A., Zhang B., Evans O. (2018). When will AI exceed human performance? Evidence from AI experts. J. Artif. Intell. Res..

[126] Kuang L., He LI U., Yili RE N., Kai LU O., Mingyu SH I., Jian S.U., Xin L.I. (2021). Application and development trend of artificial intelligence in petroleum exploration and development. Pet. Explor. Dev..

[127] Cui Y., Zhang H., Zhu J., Peng L., Duan Z., Liu T., Zuo J., Xing L., Liao Z., Wang S. (2021). Unstimulated Parotid Saliva Is a Better Method for Blood Glucose Prediction. Appl. Sci..

[128] Cui Y., Zhang H., Wang S., Lu J., He J., Liu L., Liu W. (2022). Stimulated Parotid Saliva Is a Better Method for Depression Prediction. Biomedicines.

[129] Yadan Z., Jian W., Yifu L., Haiying L., Jie L., Hairui L. (2022). Solving the inverse problem based on UPEMD for electrocardiographic imaging. Biomed. Signal. Process. Control..

[130] Cui Y., Zhang H., Zhu J., Liao Z., Wang S., Liu W. (2022). Correlations of Salivary and Blood Glucose Levels among Six Saliva Collection Methods. Int. J. Environ. Res. Public Health.

[131] Zhan X., Wang Z., Yang M., Luo Z., Wang Y., Li G. (2020). An electronic nose-based assistive diagnostic prototype for lung cancer detection with conformal prediction. Measurement.

[132] Lu C., Lemay A., Chang K., Höbel K., Kalpathy-Cramer J. Fair conformal predictors for applications in medical imaging. Proceedings of the AAAI Conference on Artificial Intelligence.

[133] Cui J., Zhu H., Deng H., Chen Z., Liu D. (2021). FeARH: Federated machine learning with anonymous random hybridization on electronic medical records. J. Biomed. Inform..

